# dsRNA-seq Reveals Novel RNA Virus and Virus-Like Putative Complete Genome Sequences from *Hymeniacidon sp.* Sponge

**DOI:** 10.1264/jsme2.ME19132

**Published:** 2020-02-28

**Authors:** Syun-ichi Urayama, Yoshihiro Takaki, Daisuke Hagiwara, Takuro Nunoura

**Affiliations:** 1 Research Center for Bioscience and Nanoscience (CeBN), Japan Agency for Marine-Earth Science and Technology (JAMSTEC), 2–15 Natsushima-cho, Yokosuka, Kanagawa 237–0061, Japan; 2 Laboratory of Fungal Interaction and Molecular Biology (donated by IFO), Department of Life and Environmental Sciences, University of Tsukuba, 1–1–1 Tennodai, Tsukuba, Ibaraki, 305–8577, Japan; 3 Microbiology Research Center for Sustainability (MiCS), University of Tsukuba, 1–1–1 Tennodai, Tsukuba, Ibaraki, 305–8577, Japan; 4 Super-cutting-edge Grand and Advanced Research (SUGAR) Program, JAMSTEC, 2–15 Natsushima-cho, Yokosuka, Kanagawa 237–0061, Japan

**Keywords:** RNA virus, metagenome, dsRNA, sponge

## Abstract

Invertebrates are a source of previously unknown RNA viruses that fill gaps in the viral phylogenetic tree. Although limited information is currently available on RNA viral diversity in the marine sponge, a primordial multicellular animal that belongs to the phylum *Porifera*, the marine sponge is one of the well-studied holobiont systems. In the present study, we elucidated the putative complete genome sequences of five novel RNA viruses from *Hymeniacidon* sponge using a combination of double-stranded RNA sequencing, called fragmented and primer ligated dsRNA sequencing, and a conventional transcriptome method targeting single-stranded RNA. We identified highly diverged RNA-dependent RNA polymerase sequences, including a potential novel RNA viral lineage, in the sponge and three viruses presumed to infect sponge cells.

The marine sponge is a primordial multicellular animal that belongs to the phylum *Porifera*. Sponges are recognized as hosts of diverse symbiotic lives (Taylor *et al.*, 2007), including bacteria, archaea, fungi, protists, and viruses (Webster and Thomas, 2016), and the relationships between the host sponge and symbionts vary from mutualism to parasitism (Webster and Taylor, 2012; Thomas *et al.*, 2016). To understand the ecosystems associated with sponges, sponges have been described as “holobionts,” which are defined as assemblages of multiple species that form ecological units (Webster and Taylor, 2012). Sponge-bacteria/-archaea interactions have been extensively examined (Schmitt *et al.*, 2012; Thomas *et al.*, 2016; Pita *et al.*, 2018); however, the impact of viruses on sponge holobionts remains unclear. Among the limited number of virus studies on sponge holobionts, the presence of diverse virus-like particles in sponge cells and symbiont compartments has been reported (Vacelet and Gallissian, 1978; Pascelli *et al.*, 2018). Furthermore, metagenomic analyses suggested the dominance of bacteriophage lineages in DNA viromes associated with reef sponge holobionts (Butina *et al.*, 2015; Laffy *et al.*, 2016; Laffy *et al.*, 2018). To the best of our knowledge, one metatranscriptome study on a sponge holobiont characterized the presence of the partial genome sequences of RNA viruses related to the families *Narnaviridae*, *Dicistroviridae*, *Partitiviridae*, *Picobirnaviridae*, *Picornaviridae*, *Tombusviridae*, *Nodaviridae*, and *Hepeviridae* as well as unclassified viruses (Waldron, *et al.* [2018] Metagenomic sequencing suggests arthropod antiviral RNA interference is highly derived, and identifies novel viral small RNAs in a mollusc and a brown alga. *bioRxiv* doi: https://doi.org/10.1101/166488).

To access RNA viromes, conventional total RNA-seq (single-stranded RNA [ssRNA]-seq) is the most commonly used method to detect RNA viral sequences in diverse environments and organisms, such as oceanic waters (Steward *et al.*, 2013; Culley *et al.*, 2014), lake waters (Djikeng *et al.*, 2009), seafloor sediments (Engelhardt *et al.*, 2015), invertebrates (Shi *et al.*, 2016), and vertebrates (Shi *et al.*, 2018). In the latter two studies, deep RNA sequencing was performed on total RNAs from invertebrates and vertebrates and thousands of RNA viruses that appeared to infect them were identified (Shi *et al.*, 2016; 2018). However, this method does not have the capacity to obtain complete genome sequences from RNA viruses because of the principle of cDNA synthesis. The rapid amplification of the cDNA end (RACE) or alternative methods have been conducted to obtain complete RNA viral genomes/segments, including terminal sequences, (Roossinck *et al.*, 2010; Shi *et al.*, 2016). In contrast, double-stranded RNA (dsRNA) sequencing has the advantage of the superior enrichment of RNA viral genomes over the conventional RNA sequencing method (Roossinck *et al.*, 2010). Long cellular dsRNA consists of dsRNA virus genomes and replicative intermediates of ssRNA viruses (Morris and Dodds, 1979). Fragmented and primer-ligated dsRNA sequencing (FLDS), a method to obtain the full-length sequence of dsRNA including terminal sequences, was recently developed (Koyama *et al.*, 2016; Urayama *et al.*, 2016; 2018b; Fukasawa *et al.*, 2019; Higashiura *et al.*, 2019). The feature of this method is to retrieve the sequences from terminal regions that are needed to reconstruct segmented RNA virus genomes based on the conserved sequences of terminal regions in each genome segment (Urayama *et al.*, 2018a). In the present study, we applied the FLDS method in combination with ssRNA-seq to reveal sponge RNA viromes.

## Materials and Methods

### Sample collection

Sponges on tidal rocks in Tokyo Bay (35.3405°N, 139.6396°E) were sampled in April 2014 and 2015. After washing with distilled water, samples were stored at –80°C until further analyses.

### Extraction and purification of dsRNA and ssRNA

Sponge samples were disrupted in liquid nitrogen in a mortar. Total nucleic acids were manually extracted from approximately 1‍ ‍g of sponge powder. dsRNA was purified using a combination of the cellulose column method (Urayama *et al.*, 2015) and an enzymatic treatment, as described previously (Urayama *et al.*, 2018a). Some of the powder was processed to obtain total RNA using the TRIzol Plus RNA Purification Kit (Invitrogen) according to the manufacturer’s protocol. Total RNA was treated with DNase I (Invitrogen) and further purified using the RNA Clean and Concentrator-5 Kit (Zymo Research).

### dsRNA sequencing library construction

Nuclease-treated dsRNA was fragmented by ultrasound at 4°C in Snap-Cap microTUBEs (Covaris) using Covaris S220 (settings: run time 35‍ ‍s, peak power 140.0 W, duty factor 2.0%, and 200 cycles burst^–1^) (Covaris) (Urayama *et al.*, 2018a). After the purification of fragmented dsRNAs using the Zymoclean Gel RNA Recovery Kit (Zymo Research), double-stranded cDNA was synthesized as described previously (Urayama *et al.*, 2018a). Briefly, a ssDNA adapter was ligated to the 3′-ends of fragmented dsRNA and cDNA was synthesized using the SMARTer RACE 5′/3′ Kit (Takara Bio) with an oligonucleotide primer that had a complementary sequence to the adapter sequence. After amplification, cDNA was quantified using the Qubit dsDNA HS Kit (Thermo Fisher Scientific) and Agilent 2100 Bioanalyzer (Agilent Technologies).

### ssRNA sequencing library construction

Double-stranded cDNA was synthesized from 2‍ ‍μg of total RNA with the Prime Script Double Strand cDNA Synthesis Kit (Takara Bio) using random primers (9-mers). The resultant cDNA was quantified using the Qubit dsDNA HS Kit (Thermo Fisher Scientific) and Agilent 2100 Bioanalyzer (Agilent Technologies).

### Illumina sequencing

Double-stranded cDNAs were fragmented using Covaris S220 (settings: run time 55‍ ‍s, peak power 175.0 W, duty factor 5.0%, and 200 cycles burst^–1^), and fragmented cDNAs were used as templates for KAPA Hyper Prep Kit Illumina platforms (Kapa Biosystems). The resultant Illumina libraries were evaluated using the KAPA library quantification kit (Kapa Biosystems) and applied to the Illumina MiSeq platform (Illumina) according to the manufacturer’s protocol (600-cycle kit to perform 300-bp paired-end sequencing).

### Data processing

Raw sequencing reads for dsRNA-seq were processed as described previously (Urayama *et al.*, 2018a). rRNA reads in dsRNA-seq libraries were identified by SortMeRNA (Kopylova *et al.*, 2012) and excluded from further virome analyses. In RNA virome analyses, reads were subjected to *de novo* assembly using CLC Genomics Workbench ver. 11.0 (CLC Bio) with the following parameters: a minimum contig length of 500, word value and bubble size set to auto, and assemblies that were manually examined and extended using the Tablet viewer (Milne *et al.*, 2010). Using the mapping tool, each contig was screened under the following conditions: at least 3× average coverage and 500 bp in length. If >10 reads defined the same terminal position of a certain contig, the position was recognized as a terminal end. Contigs for which both ends were identified as termini were defined as potential genome segments. RNA viral genes in potential genome segments and contigs were identified based on sequence similarities to known RNA viral proteins in the NCBI non-redundant (nr) database using BLASTX (Camacho *et al.*, 2009) with an e-value≤1×10^–5^. Sequences that matched a known RNA-dependent RNA polymerase (RdRp) gene by BLASTX with an e-value≤1×10^–5^ were collected from RNA virus contigs and segments. The presence of the RdRp domain (PF00680, PF00978, and PF02123) was confirmed using PfamScan (Chojnacki *et al.*, 2017) with an e-value≤1×10^–5^ when a sequence had significant similarity (e-value≤1×10^–5^) to an RNA viral polyprotein or hypothetical protein. Nucleotide sequences encoding the RdRp gene were clustered at 90% identity using VSEARCH (Rognes *et al.*, 2016). The centroid sequence of each cluster was defined as a representative sequence. We also identified the signature amino acid residues conserved in the RdRp domain (Venkataraman *et al.*, 2018) in the ORFs of potential genome segments that had no ORF with significant similarities (e-value≤1×10^–5^) to sequences in the nr database. Quality-based variant detection was performed using CLC Genomics Workbench ver. 11.0 (CLC Bio).

Raw sequencing reads from ssRNA-seq were processed as described previously (Urayama *et al.*, 2016). Small subunit (SSU) rRNA sequences were reconstructed from ssRNA-seq reads with EMIRGE (Miller *et al.*, 2011).

### Phylogenetic analyses

The deduced amino acid sequences of the putative RdRp genes obtained in the present study and their relatives in the NCBI nr database were aligned using MUSCLE (Edgar, 2004) in MEGA5 (Tamura *et al.*, 2011). To exclude ambiguous positions, the alignment was trimmed by trimAl (option: -gt 1) (Capella-Gutiérrez *et al.*, 2009). Phylogenetic analyses were conducted using RAxML (Stamatakis, 2014). Bootstrap tests were conducted with 1,000 samplings. The model of amino acid substitution for each phylogeny was selected by Aminosan (Tanabe, 2011), as judged by Akaike’s information criterion (Sugiura, 1978), *i.e.*, rtREV+F+G for *Partitiviridae*, LG+F for *Reoviridae*, LG+F+G for *Picobirnaviridae*, and rtREV+F+G for *Dicistroviridae*. TreeGraph2 (Stöver and Müller, 2010) and FigTree (Rambaut, 2014) were used to illustrate the resulting phylogenies.

### Accession numbers

The sequences obtained in the present study are available in the GenBank database repository (accession nos. DDBJ: BKZT01000001–BKZT01000434, BKZU01000001–BKZU01000431, and LC504241–LC504250) and Short Read Archive database (accession nos. DDBJ: DRA008491).

## Results

### Overview of NGS reads

We obtained 1.8–4.1 M high-quality reads in four RNA-seq libraries (Fig. S1). Regarding dsRNA-seq libraries, reads associated with RNA viruses (see below) and the remaining rRNA reads occupied approximately 20 and 30% of dsRNA reads, respectively. The ratio of rRNA reads in dsRNA-seq libraries varied among the samples reported previously (Urayama *et al.*, 2016; 2018a; 2018b; Decker *et al.*, 2019). Some rRNAs potentially contaminated the dsRNA fraction due to their secondary structures. Thus, the relative amount of dsRNA to rRNA in samples may have affected the rRNA read ratio in dsRNA sequencing. Furthermore, the formation of dsRNAs of sense/antisense transcripts from overlapping regions may influence the results of dsRNA sequencing (Decker *et al.*, 2019). In ssRNA-seq libraries, RNA viruses and rRNA reads occupied approximately 0.5 and 80% of ssRNA reads, respectively.

### Diversity of cellular rRNA in sponges

**Fig. 1.**
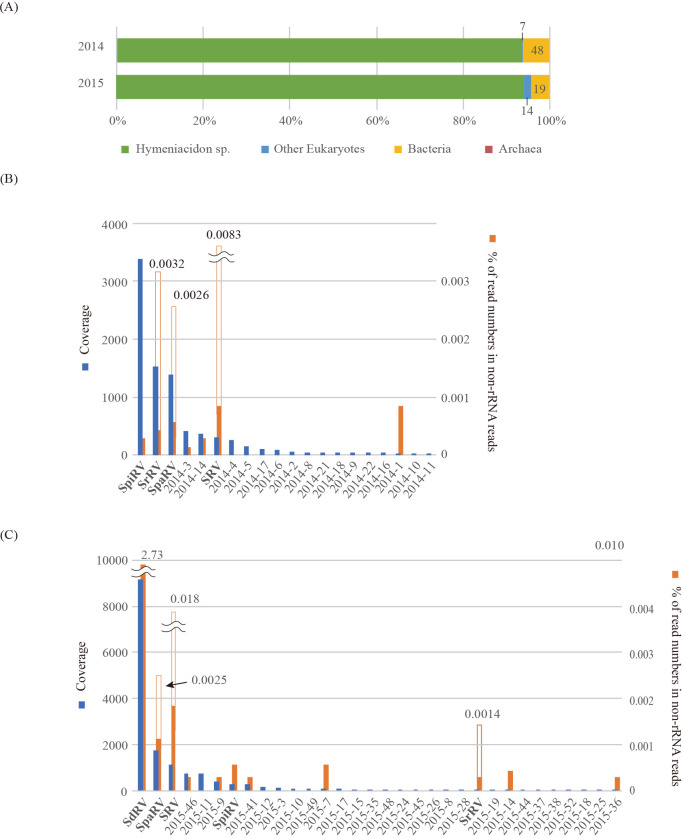
Summary of dsRNA-seq and ssRNA-seq analyses. (A) Relative abundance of sequence reads mapped on SSU rRNA sequences constructed by EMIRGE. Numbers indicate OTU. (B, C) The average coverage (left axis) and relative abundance (right axis) of RdRp-encoding RNA viral genome segments (Table S1) estimated by the mapping of dsRNA-seq and ssRNA-seq data, respectively, from sponge samples collected in 2014 (B) and 2015 (C). Open bars indicate reads mapped on segments encoding CDS with no significant similarity (e-value≤1×10^–5^) to known viral genes. Items on the horizontal axis represent the IDs of RdRp-encoding full-length sequences (Table S1) or virus names.

To identify potentially active biomes in sponges obtained in 2014 and 2015, SSU rRNA sequences were reconstructed from total ssRNA-seq reads, and compositions were estimated based on mapped read numbers on SSU rRNA sequences (Miller *et al.*, 2011). *Hymeniacidon sp.* SSU rRNA reads occupied >90% of all SSU rRNA reads in total ssRNA libraries from sponges obtained in both 2014 and 2015 (Fig. 1A). The next abundant eukaryotic SSU rRNA sequence was identified as *Mytilus edulis* (0.8%) mussel, and the abundance of other eukaryotic SSU rRNA sequences was lower than 0.2% of all SSU rRNA reads. No archaeal or bacterial SSU rRNA sequence was found from reads that dominated >2% of all SSU rRNA reads.

### Sponge-associated non-retro RNA virome

**Fig. 2.**
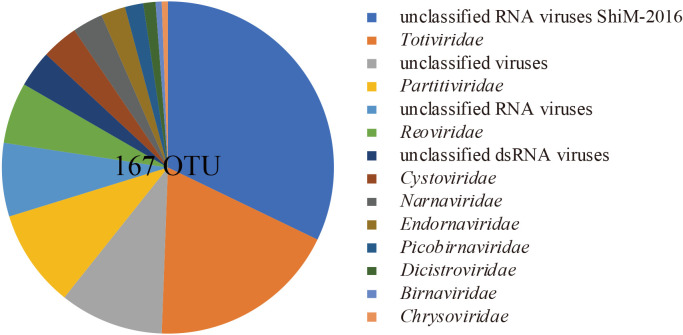
Overview of the RNA virome identified from the sponge. Taxonomic composition of RdRp-based OTUs identified in the present study.

The FLDS analysis (dsRNA-seq) succeeded in enriching viral reads and revealed RNA viromes associated with sponges collected in 2014 and 2015, while RNA virus reads were rare in the ssRNA-seq analysis, except for reads related to *Dicistroviridae* in 2015 samples (Table S1). We obtained 253 and 233 sequences encoding RdRp genes from the 2014 and 2015 samples, respectively (Table S1). These sequences included viral sequences related to 9 dsRNA virus families and 3 positive-sense ssRNA virus families based on a RdRp sequence similarity analysis, while 11 and 33 families were previously established in dsRNA and positive-sense ssRNA viruses, respectively (King *et al.*, 2012). We also found 78 sequences encoding RdRp genes belonging to unclassified RNA viral lineages based on the taxonomic linage of BLASTX top hit sequences (Table S1), and 7.7% of RdRp reads in dsRNA libraries were mapped to these segments and contigs. Viral sequences related to families recognized as animal viruses, such as *Birnaviridae* and *Dicistroviridae*, were also identified from sponge RNA viromes. After the clustering of RdRp domain sequences, 167 representative sequences (>1.5‍ ‍kb, <90% identity) were identified and defined as operational taxonomic units (OTUs). Based on the taxonomic lineage of BLASTX top hit sequences, the richness of OTUs was analyzed and many viral OTUs were suggested to be closely related with (or belonging to) *Totiviridae*, *Reoviridae*, and *Partitivirdae* (Fig. 2). The majority of OTUs in unclassified groups showed low amino acid sequence identity to the best hit sequence (Fig. S2). All of the top hit sequences in “unclassified RNA viruses” were RdRp identified from *Bryopsis cinicola* (Chlorophyta) (Koga *et al.*, 2003). In “unclassified dsRNA viruses” and “unclassified viruses”, several OTUs showed close sequence similarities to RdRp sequences identified from the Diatom colony (Urayama *et al.*, 2016). Dominance in the coverage of these dsRNA viruses was also confirmed based on the read abundance in dsRNA libraries (Table S1).

### Major putative complete genomes

**Fig. 3.**
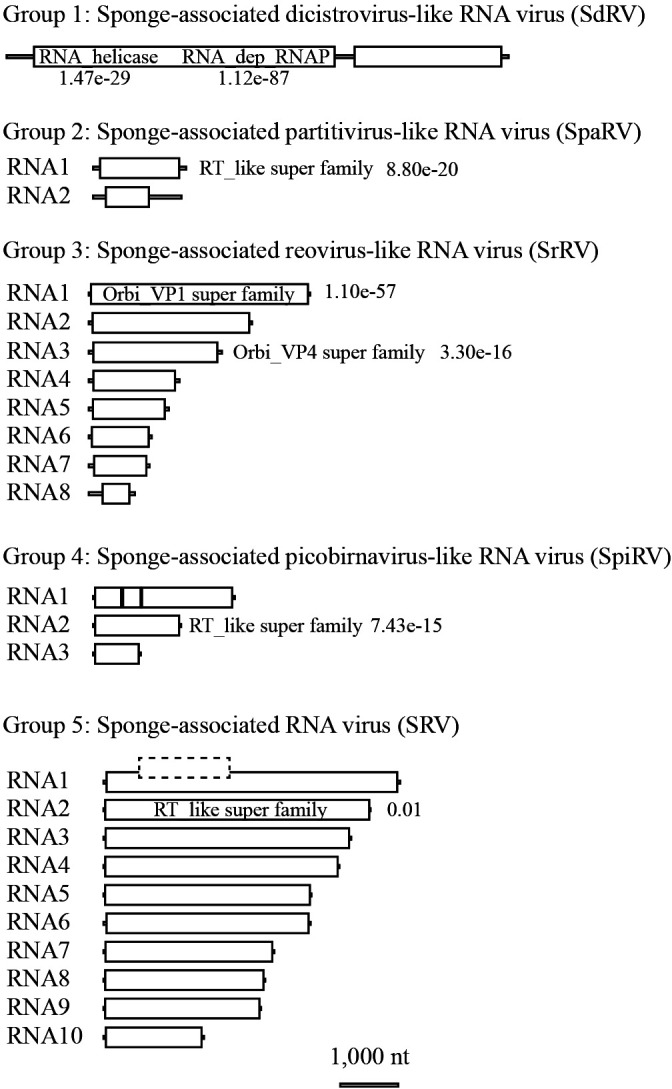
Organization of reconstructed RNA viral or virus-like genomes. Values indicate the e-value with the viral top hit domain by a CD search.

We obtained 118 and 180 full-length potential viral genome segments from 2014 and 2015 samples, respectively. Among RdRp-encoding potential viral genome segments (Fig. 1B and C), we focused on the five dominant viral genome segments that represented high average coverage (>1,000×) in each dsRNA-seq library. The five segments occupied more than 50% of the total average coverage of RdRp-encoding segments and contigs (253 and 233 OTU in 2014 and 2015, respectively). Following the identification of major RdRp sequences in dsRNA-seq libraries, we reconstructed RNA genome sets based on the similarity of terminal sequences among segments (Fig. 3 and S3). These five genome sets were considered to be “putative complete” because they had very high coverage (>1000×), and, thus, there was a low chance of missing other fragments of their genomes. Reconstructed viral genomes were also supported by the following parameters, in addition to the similarity of terminal sequences. The difference in GC% among sequences within a group sharing similar terminal sequences was <5%. Each ORF did not show amino acid sequence similarity (>50% similarity) to any ORFs encoded by other potential genome segments in a single group. We named these RNA viral genomes harboring ORFs that presented significant similarity (e-value≤1×10^–5^) with known viral sequences after the names of the most closely related virus family (Table S2 and see below); Sponge-associated dicistro-like RNA virus (SdRV, accession no. BKZU01000024), Sponge-associated partiti-like RNA virus (SpaRV, accession no. BKZT01000015), Sponge-associated reo-like RNA virus (SrRV, accession no. BKZT01000005), Sponge-associated picobirna-like RNA virus (SpiRV, accession no. BKZT01000016), and Sponge-associated RNA virus (SRV, accession no. LC504241–50).

No ORF of SRV showed significant similarity (e-value≤1×10^–5^) with sequences in the nr database. However, an ORF of SRV RNA2 showed insignificant similarity (e-value=0.01) to the RT_like super family (cl02808) in the CDD search. Thus, we manually identified the signature amino acid residues conserved in the RdRp domain (Venkataraman *et al.*, 2018) including the “GDD” sequence in the ORF (Fig. S4). Accordingly, we concluded that the genome segment set corresponded to RNA viral genomes that belonged to a previously unknown viral family.

To estimate the potential host of these RNA viruses identified in the dsRNA-seq analysis, we evaluated the abundance of these viral sequences in ssRNA-seq according to the previously proposed definition that viral reads sharing >0.1% in non-rRNA reads in conventional RNA sequencing may be infected with the sampled organism (Shi *et al.*, 2016). Among the five major viral genomes, SdRV showed the highest read abundance, accounting for 2.7% of non-ribosomal reads in the ssRNA-seq library (Fig. 1C). The full-length sequence of SpaRV was obtained in 2014 and 2015 samples and only one base substitution was observed between sequences obtained from the two samplings. The read abundance in non-ribosomal reads was approximately 0.0025% in both ssRNA-seq libraries. SRV was also detected in 2014 and 2015 samples and occupied more than 0.008% in both ssRNA-seq libraries. Between the 2014 and 2015 SRV genomes, there were only 54 positions of a single-nucleotide variant and 90% (49/54) of them were identified as transitions. Therefore, SpaRV and SRV appeared to have infected the same or closely related organisms (host) within sponges obtained in both 2014 and 2015. In contrast, in both sponges, eukaryotes other than *Hymeniacidon* sponge did not dominate more than 0.0025% of the SSU rRNA composition, with the exception of *M. edulis* in 2015 samples, which shared 0.8% of SSU rRNA reads. Thus, we concluded that *Hymeniacidon sp.* appeared to be a host of SdRV, SpaRV, and SRV. The read abundances of SpiRV and SrRV putative genomes were one to two orders of magnitude smaller than that of SdRV in ssRNA-seq data, and were only detected from either of the samples. Therefore, we were unable to define their host based on this definition. It is important to note that dsRNA viruses (SpaRV, SpiRV, and SrRV) showed lower read abundance than the ssRNA virus (SdRV) in ssRNA-seq data.

### SdRV

**Fig. 4.**
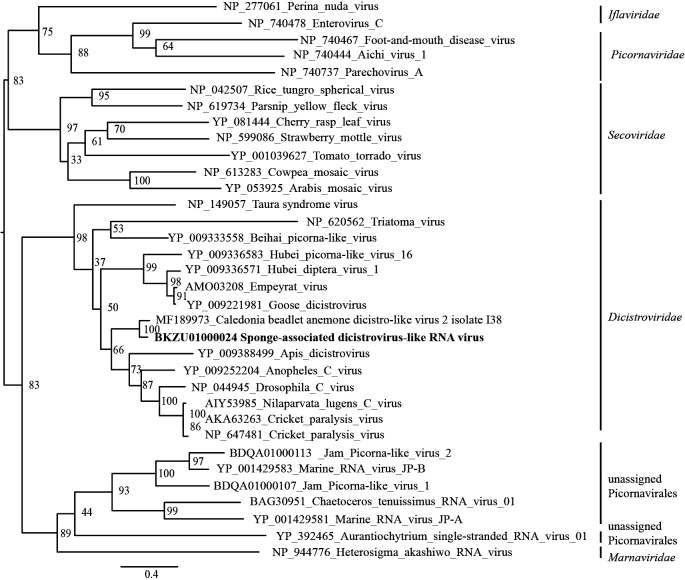
Maximum likelihood tree of aligned RdRp amino acid sequences from SdRV and representative members of families *Dicistroviridae*, *Secoviridae*, *Picornaviridae*, and *Iflaviridae* using 305 residues. Numbers indicate bootstrap values (%) based on 1,000 RAxML calculations. The best-fitting amino acid substitution model was [rtREV+F+G].

Among reconstructed viral genomes, only SdRV presented significant similarity with the subject sequence Caledonia beadlet anemone dicistro-like virus 2 isolate I38 (accession no. MF189973, publication status: unpublished) (e-value=0.0, 71% identity) in the BLASTN analysis; its potential host Caledonia belongs to the phylum *Cnidaria*, not *Porifera*. The SdRV genome consists of one segment with two ORFs. The BLASTP search also revealed that the most similar sequence to SdRV RdRp was putative ORF1 of Caledonia beadlet anemone dicistro-like virus 2 isolate I38 (accession no. ASM93984) (e-value=0.0, 74% identity). A phylogenetic analysis of the RdRp sequence indicated that SdRV belonged to the positive-stranded ssRNA virus family *Dicistroviridae* (Fig. 4). The arrangement of ORFs on the SdRV genome was similar to those on known viruses in *Dicistroviridae* (Fig. S5). These results suggest that SdRV is a new ssRNA virus that belongs to *Dicistroviridae*.

### SpaRV

**Fig. 5.**
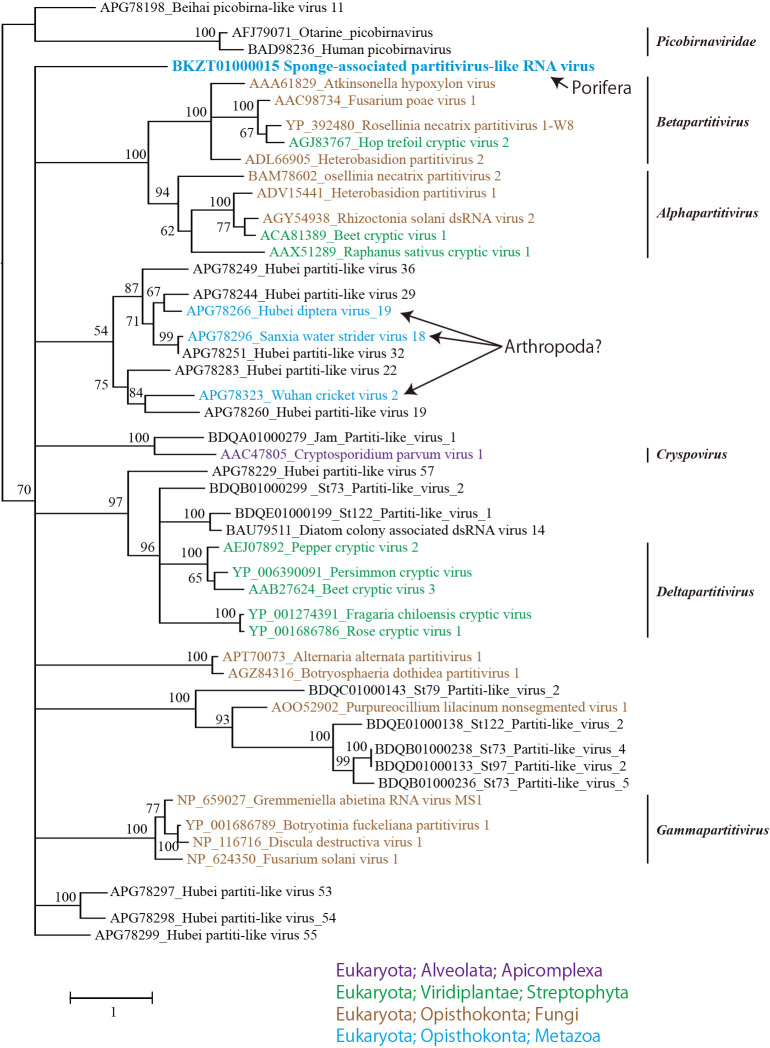
Maximum likelihood tree of aligned RdRp amino acid sequences from SpaRV and representative members of families *Picobirnaviridae* and *Partitiviridae* using 302 residues. Numbers indicate bootstrap values (%) based on 1,000 RAxML calculations. The best-fitting amino acid substitution model was [rtREV+F+G]. Colors indicate the classification of the host organism.

In the BLASTP analysis for RdRp in the SpaRV genome, the most similar sequence in the nr database was Hubei partiti-like virus 53 (related to viruses in *Partitiviridae*) (e-value=3×10^–43^, 32% identity) found in the ssRNA-seq of invertebrates (Shi *et al.*, 2016). The genome of SpaRV consists of two segments with one ORF each and the genome structure is conserved in known partitiviruses. Although the RdRp sequence showed significant similarity (e-value≤1×10^–5^) with sequences in *Partitiviridae*, SpaRV was not classified into the established genus of *Partitiviridae* by the phylogenetic analysis (Fig. 5) (Nibert *et al.*, 2014). Therefore, SpaRV represents a novel genus in the family *Partitiviridae*. Viruses in *Partitiviridae* have been isolated from culturable organisms, such as plants, fungi, and protozoa (Nibert *et al.*, 2014). In addition, their relatives have been detected in both vertebrates and invertebrates in transcriptomic analyses (Liu *et al.*, 2012; Shi *et al.*, 2016); however, no partitivirus has been isolated from animals (Nibert *et al.*, 2014).

### SrRV, SpiRV, and SRV

**Fig. 6.**
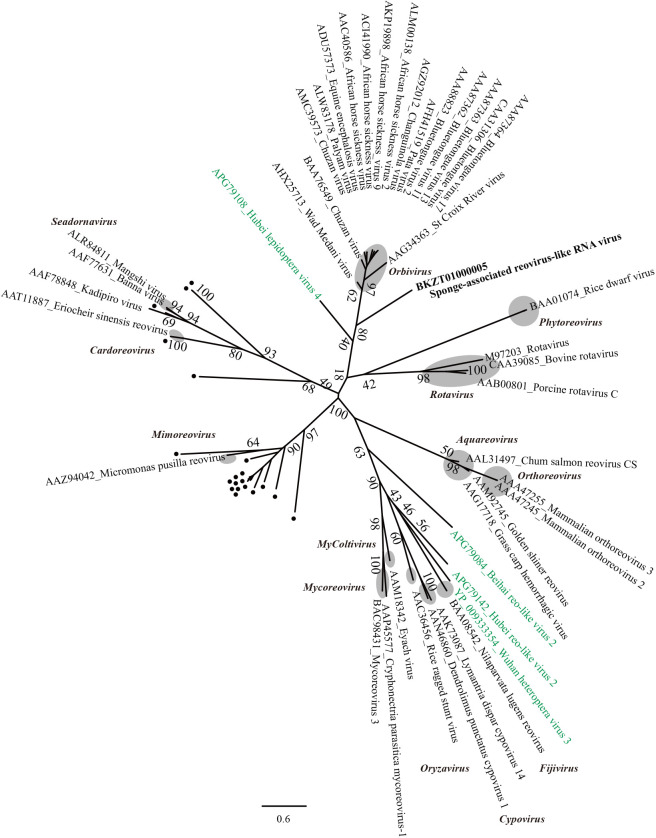
Maximum likelihood tree of aligned RdRp amino acid sequences from SrRV and representative members of the family *Reoviridae* using 409 residues. Numbers indicate bootstrap values (%) based on 1,000 RAxML calculations. The best-fitting amino acid substitution model was [LG+F]. Gray circles represent established genera. Black dots and green color represent OTUs derived from Urayama *et al.* (2018a) and Shi *et al.* (2016), respectively.

The sequence that was the most similar to RdRp in SrRV in the BLASTP analysis was Chuzan virus (genus *Orbivirus*) (e-value=2×10^–14^, 25% identity); however, a phylogenetic analysis of RdRp for *Reoviridae* revealed that SrRV was not positioned into the genus *Orbivirus* (Fig. 6). In addition, in the BLASTX analysis, an ORF of segment 3 showed significant similarity with VP3 (Orbivirus VP4 core protein) of Wad Medani virus (genus *Orbivirus*) (e-value=2×10^–14^, 25% identity), while other segments showed no significant similarity (e-value≤1×10^–5^) with known viral sequences.

**Fig. 7.**
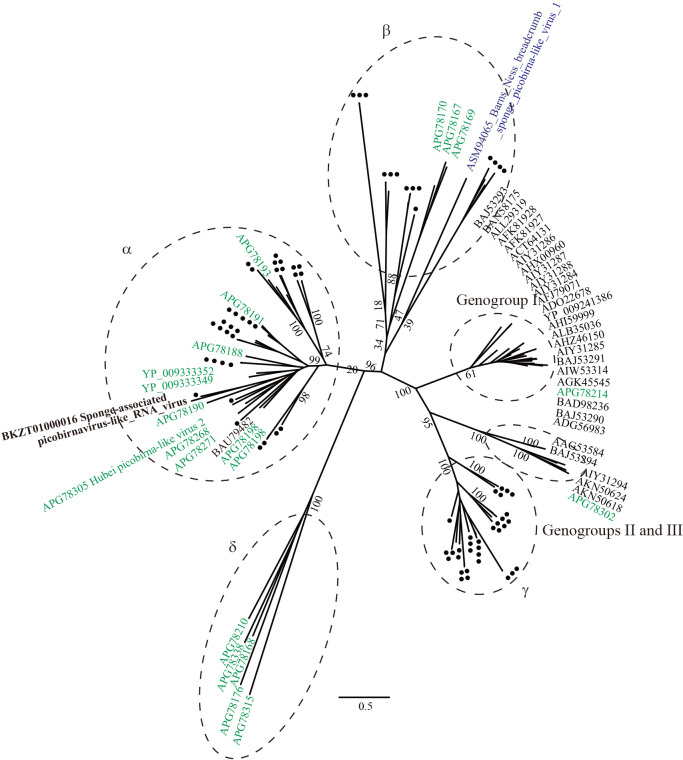
Maximum likelihood tree of aligned RdRp amino acid sequences from SpiRV and representative members of the family *Picobirnaviridae* using 310 residues. Numbers indicate bootstrap values (%) based on 1,000 RAxML calculations. The best-fitting amino acid substitution model was [LG+F+G]. Black dots and green color represent OTUs derived from Urayama *et al.* (2018b) and Shi *et al.* (2016), respectively. Purple color indicates unpublished deposited sequences.

The most significantly similar sequence in the BLASTP analysis for RdRp in SpiRV was Hubei picobirna-like virus 2. A phylogenetic analysis of RdRp suggested that SpiRV belongs to an undefined and tentatively named α clade of picobirnavirus-like viruses derived from marine microorganisms, diatoms, and invertebrates (Urayama *et al.*, 2018a) (Fig. 7). In *Picobirnaviridae*, one sequence that originates from a sponge named Barns Ness breadcrumb sponge picobirna-like virus 1 (accession no. MF190026.1) was found in the nr database, but belongs to the β clade.

SRV was composed of 10 RNA segments with 35,412 nt that showed no significant similarity (e-value≤1×10^–5^) with sequences in the nr database in BLASTN and BLASTX analyses. The RdRp sequence was too distant from all known RdRp sequences to identify its phylogenic position. However, as discussed above, the genome structure of SRV resembled that of *Reoviridae* and the ratio of SRV reads in non-rRNA ssRNA-seq was similar to those of dsRNA viruses (Fig. 1B and C). When considering the maximum segment number of known ssRNA viruses (viruses in Orthomyxoviridae have ~8 segments), SRV may be a reo-like novel dsRNA virus. To the best of our knowledge, the genome size of SRV is the largest among known RNA viruses.

### Other viral segments and contigs

Although we focused on numerically abundant viruses with putative complete genome sequences in the dsRNA-seq libraries, there was a RdRp-encoding contig with similar average coverage (1,839×) to these five genomes. The contig (ID: Sponge_RNAV429) was identified in 2014 samples, and showed significant similarity (e-value=4×10^–14^, 24% identity) to known, but unclassified RdRp identified from the chloroplasts of *B. cinicola*. Other RdRp-encoding segments and contigs were lower than 1,000× average coverage.

In the present study, 788 partial viral genome sequences were identified (Table S1). To clarify the phylogenetic distribution of these viruses, maximum likelihood trees for each established RNA virus family were calculated based on RdRp sequences encoded in the contigs of partial or putative complete genome segments (Fig. S6). Consistent with the similarity values obtained by the BLASTX analysis (Table S1), most viruses were phylogenetically distinct from known RNA viral genera, and RNA viruses related to the families *Partitiviridae*, *Reoviridae*, and *Totiviridae* were very diverse.

## Discussion

Recent RNA virome analyses suggest the extent of gene module shuffling among diverse viral genomes (Dolja and Koonin, 2017). Thus, high-quality information on RNA viral genomes is essential for understanding the diversity and evolution of RNA viruses. However, the conventional metagenomic ssRNA-seq approach has a limitation in retrieving entire genomic structures because of the absence of the terminal sequences of each genome segment (Ozsolak and Milos, 2011). Most contigs encoding viral genes, except for RdRp genes, must be overlooked, except when the genome sequence presents significant similarity to previously reported viral genomes. For example, among the five possible RNA viral genomes from the two sponges examined in the present study, it was not possible to identify >50% of segments as RNA viral genome segments based on a sequence similarity analysis when the terminal sequence information of RNA viral segments obtained by FLDS was absent.

In FLDS analyses, we were unable to identify the polyA tail of SdRV; however, most viruses in the order *Picornavirales*, including the family *Dicistroviridae*, are known to harbor a polyA tail on their genomes (King *et al.*, 2012). This difference may be explained by the methodological bias of the FLDS method that targets cellular dsRNA, a replicative intermediate of ssRNA viruses (York and Fodor, 2013). A previous study reported that RNA viral genome sequences related to viruses in *Hypoviridae* (order: unassigned) with polyA tails were retrieved by FLDS (Urayama *et al.*, 2018a). The variance of the 3′ terminal modification with a polyA tail among ssRNA viral genomes retrieved by FLDS implies variations in replication processes among ssRNA viruses.

In the present study, we assessed sponge RNA viromes using dsRNA- and ssRNA-seq and identified 167 RdRp genes, which represent RNA viral OTUs. The present results revealed that RNA viruses in sponge holobionts are highly diverse, similar to DNA viruses and prokaryotic populations (Taylor *et al.*, 2004; Laffy *et al.*, 2018). The present study suggests the importance of RNA viruses for the homeostasis and evolution of sponge holobiont systems, while RNA viromes have been overlooked in holobiont systems. A high-quality list of sponge-associated RNA viruses will be a fundamental knowledge base for understanding the activity and ecological distribution of (sponge-associated) RNA viruses.

## Citation

Urayama, S., Takaki, Y., Hagiwara, D., and Nunoura, T. (2020) dsRNA-seq Reveals Novel RNA Virus and Virus-Like Putative Complete Genome Sequences from *Hymeniacidon sp.* Sponge. *Microbes Environ ***35**: ME19132.

https://doi.org/10.1264/jsme2.ME19132

## Supplementary Material

Supplementary Material 1

Supplementary Material 2
