# Supplementary material for: dsRNA-seq Reveals Novel RNA Virus and Virus-Like Putative Complete Genome Sequences from Hymeniacidon sp. Sponge
Source: Microbes Environ. 2020 Feb 28;35(2):ME19132. doi: 10.1264/jsme2.ME19132 (PMC7308569; doi:10.1264/jsme2.ME19132)
Supplement: Supplementary file 1 — Supplementary Material 1 [file 35_19132_s1.pdf]

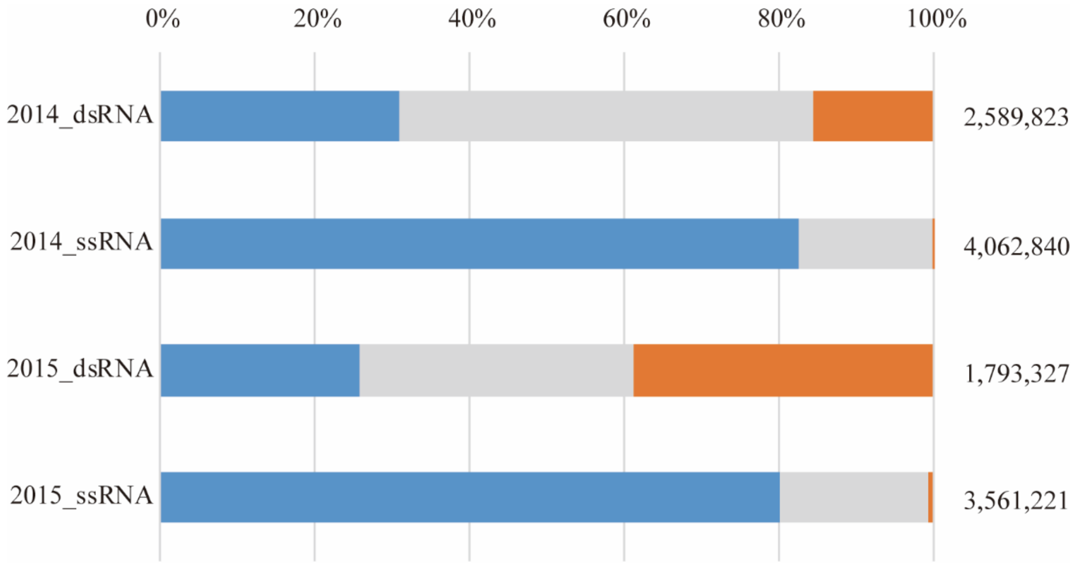

Fig. S1. Origins of NGS reads. Blue, rRNA reads; orange, five major RNA viral reads; gray, others. Numbers on the right side represent total trimmed reads.

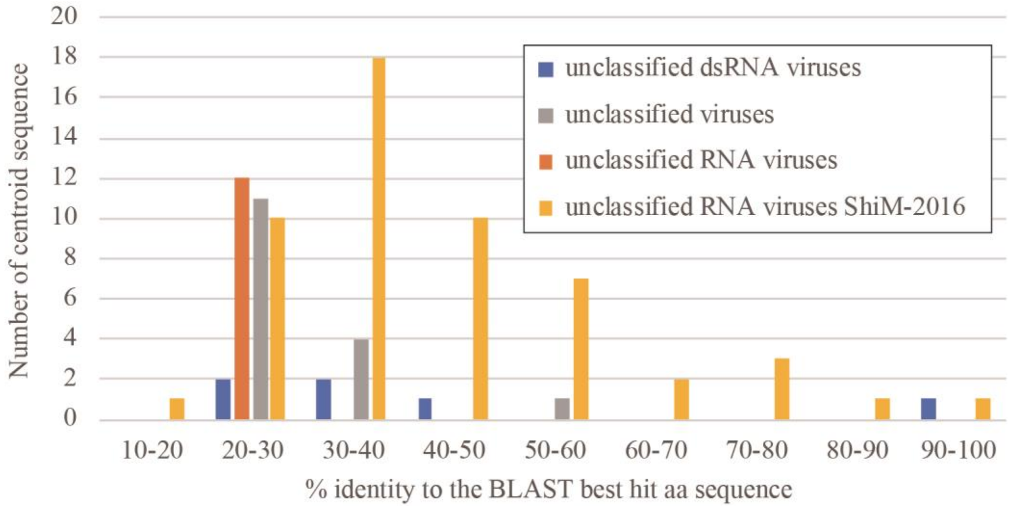

Fig. S2. Histogram of % identity for each centroid encoding RdRp to known viral aa sequence.

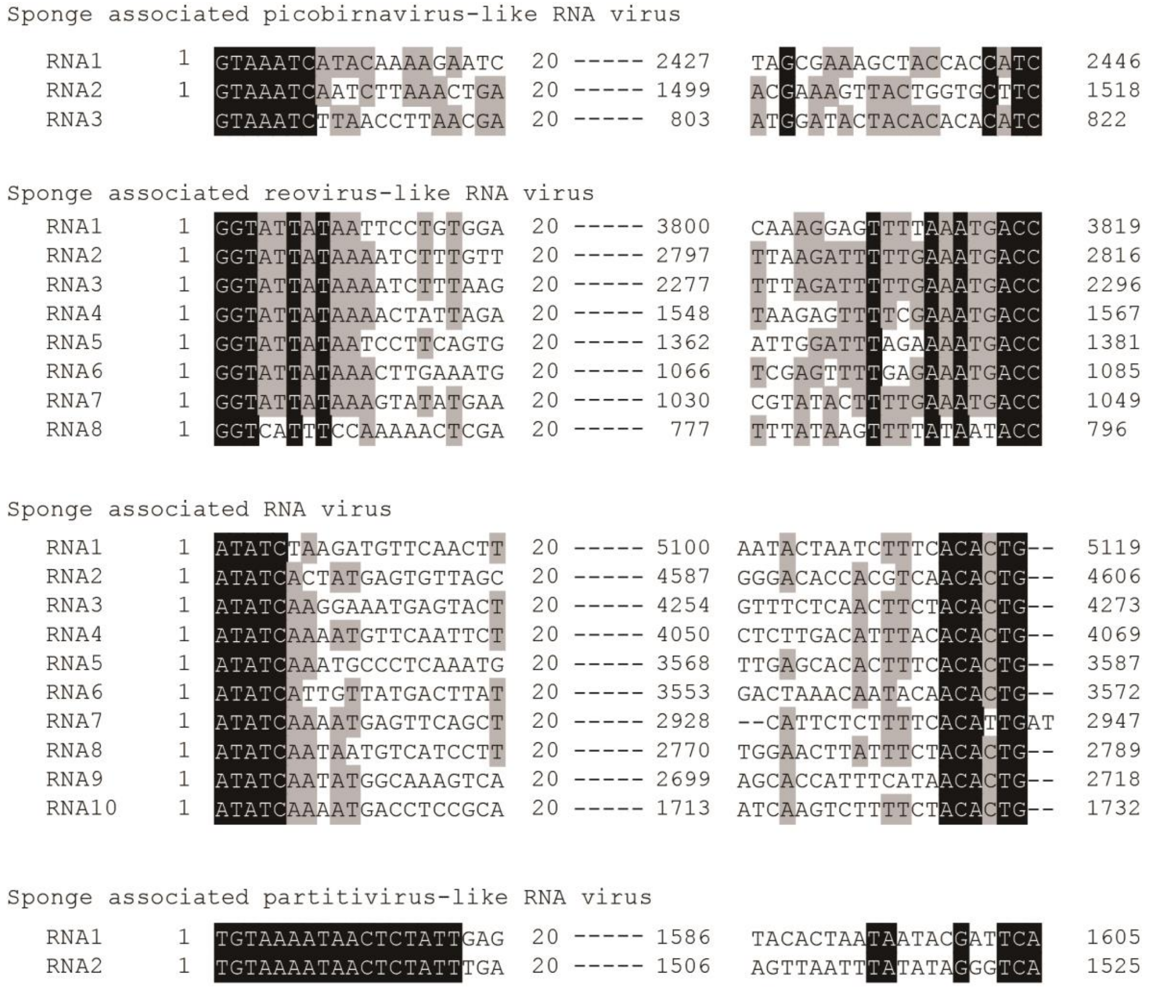

Fig. S3. Multiple alignments of the 5′- and 3′-terminal regions of the coding strands of reconstructed genome segments comprising dominant RNA viral genomes found in this study. Black shading, 100% nucleotide identity; gray shading, > 50% nucleotide identity.

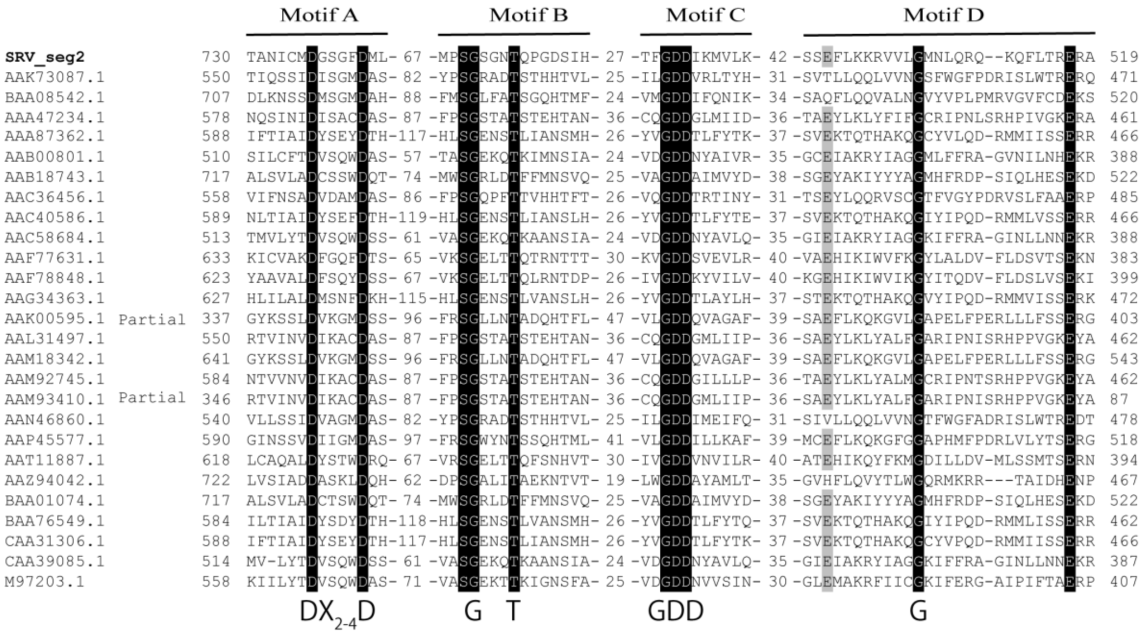

Fig. S4. Multiple alignment of the deduced amino acid sequence of the RdRp region encoded by SRV segment 2 with those of viruses in the family *Reoviridae*. Conserved motifs in RdRps are shown as motifs A–D (Venkataraman et al., 2018). Important amino acid residues found in SRV are shown at the bottom of the alignment. Black shading, 100% amino acid identity; gray shading, > 80% amino acid identity. Numbers at the beginning and end of sequences represent amino acid positions from the start of the predicted gene product.

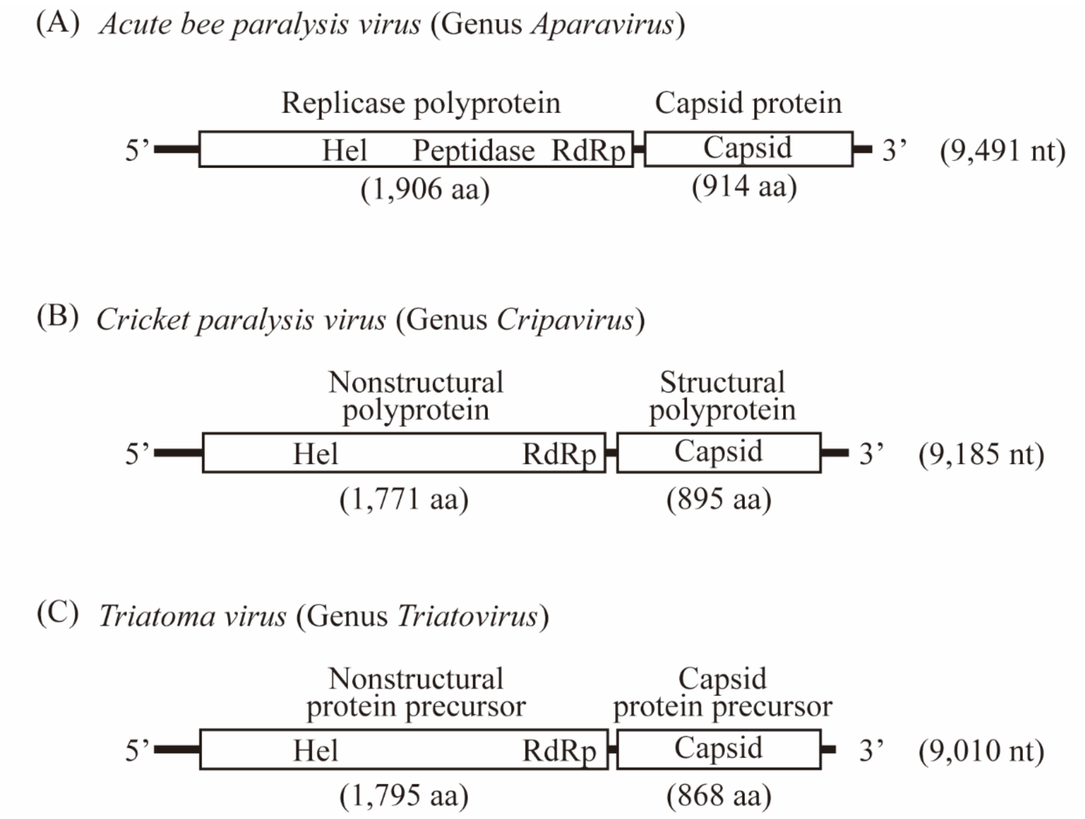

Fig. S5. Genome organization of known viruses in *Dicistroviridae*. (A) *Acute bee paralysis virus*, (B) *Cricket paralysis virus* and (C) *Triatoma virus*. Domains: Hel, Viral_helicase1 superfamily; RdRp, RNA-dependent RNA polymerase.

(A)

Birnaviridae
Amino acid substitution model: RtREV+I+G+F
Residues: 261

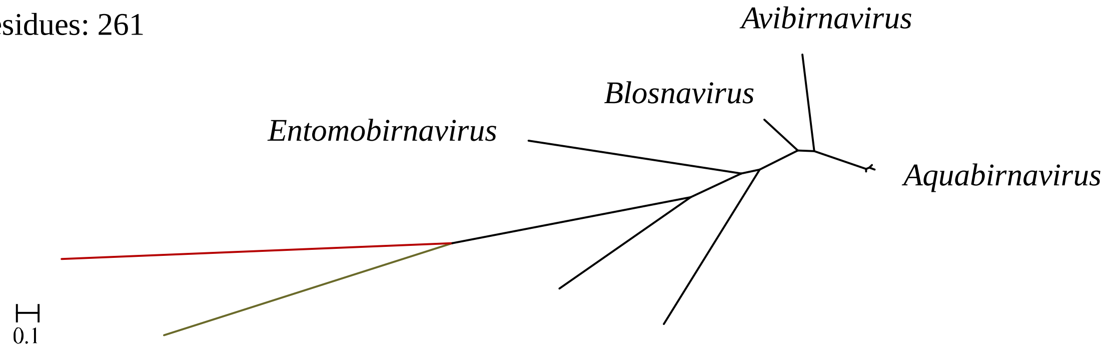

(B)

Chrysoviridae
Amino acid substitution model: LG+I+G+F
Residues: 493

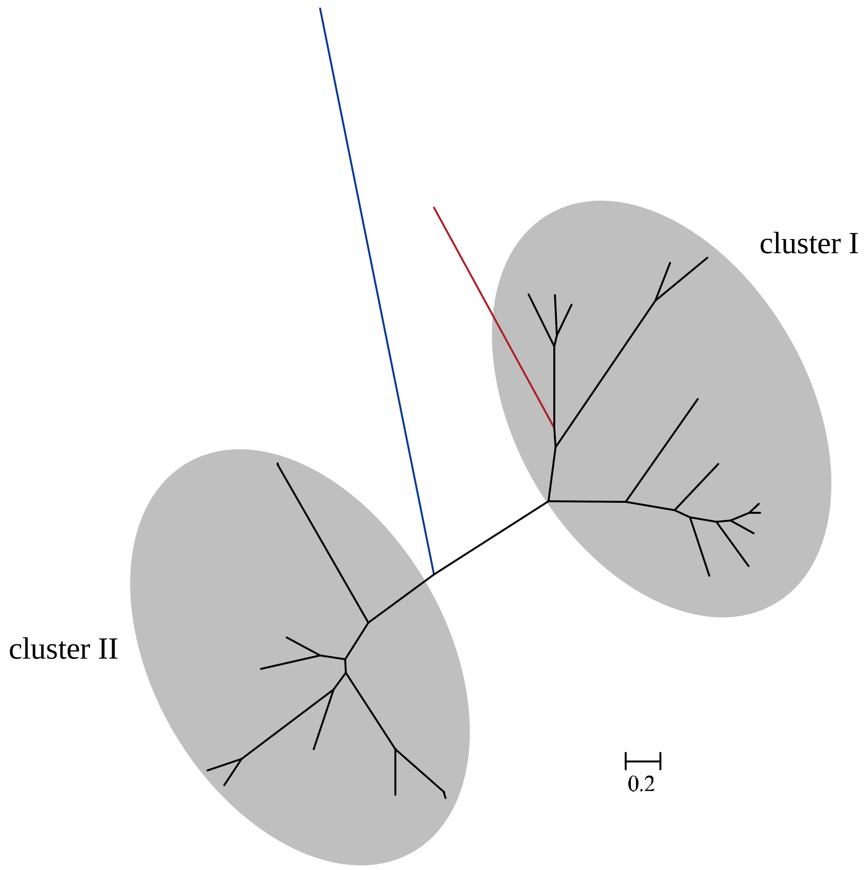

(C)

Cystoviridae
Amino acid substitution model: LG+I+G+F

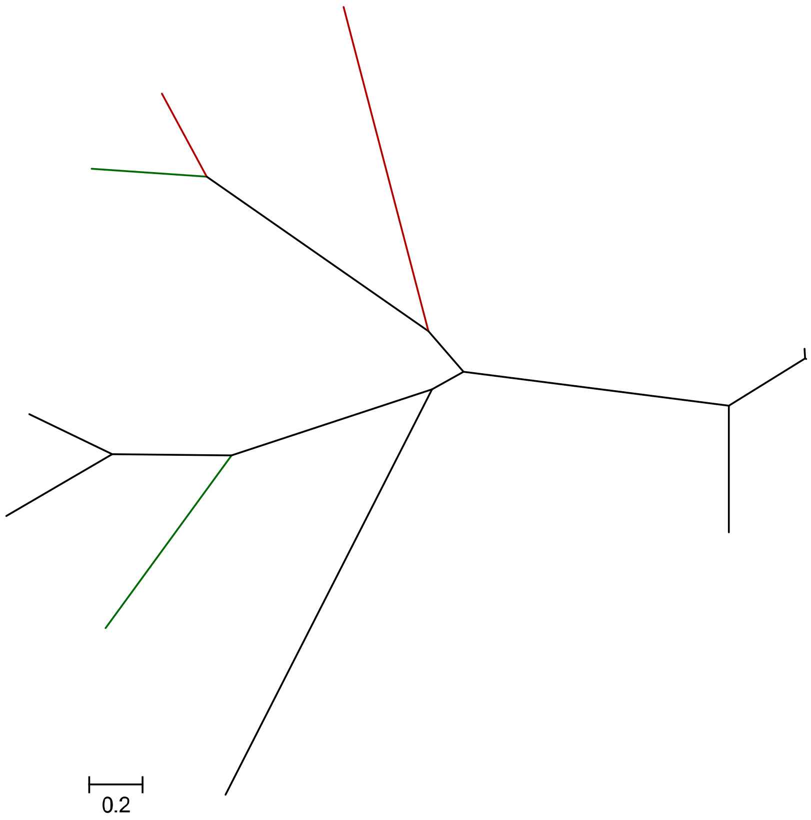

(D)

Dicistroviridae
Amino acid substitution model: LG+I+G+F
Residues: 499

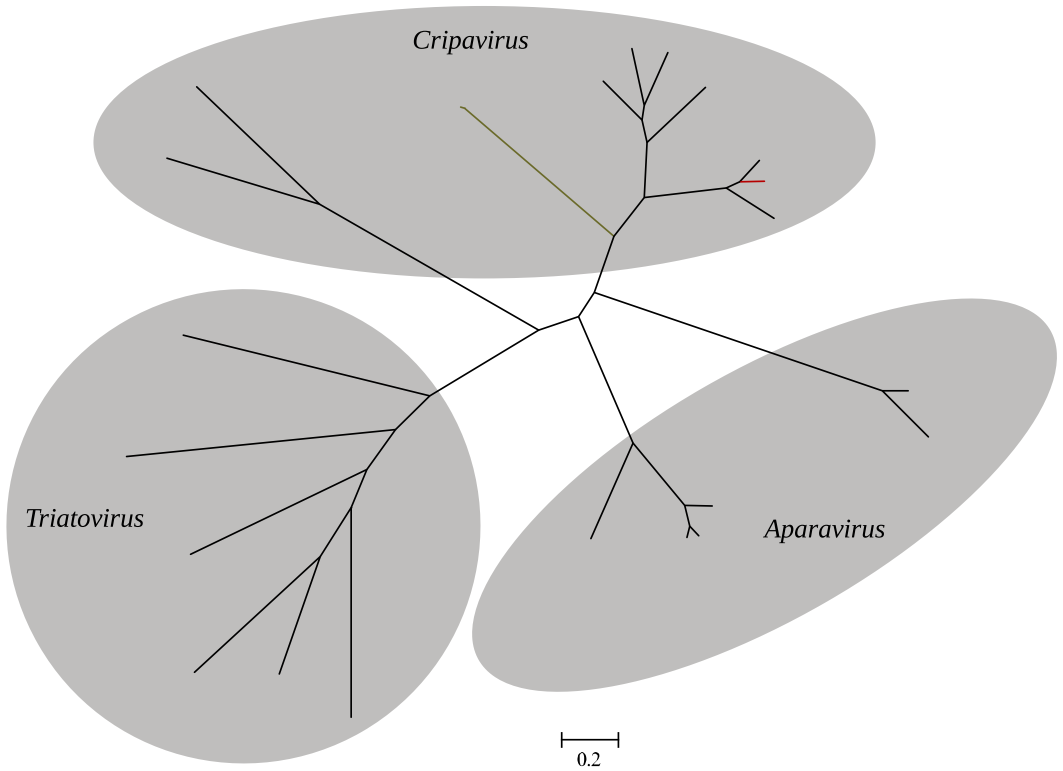

(E)

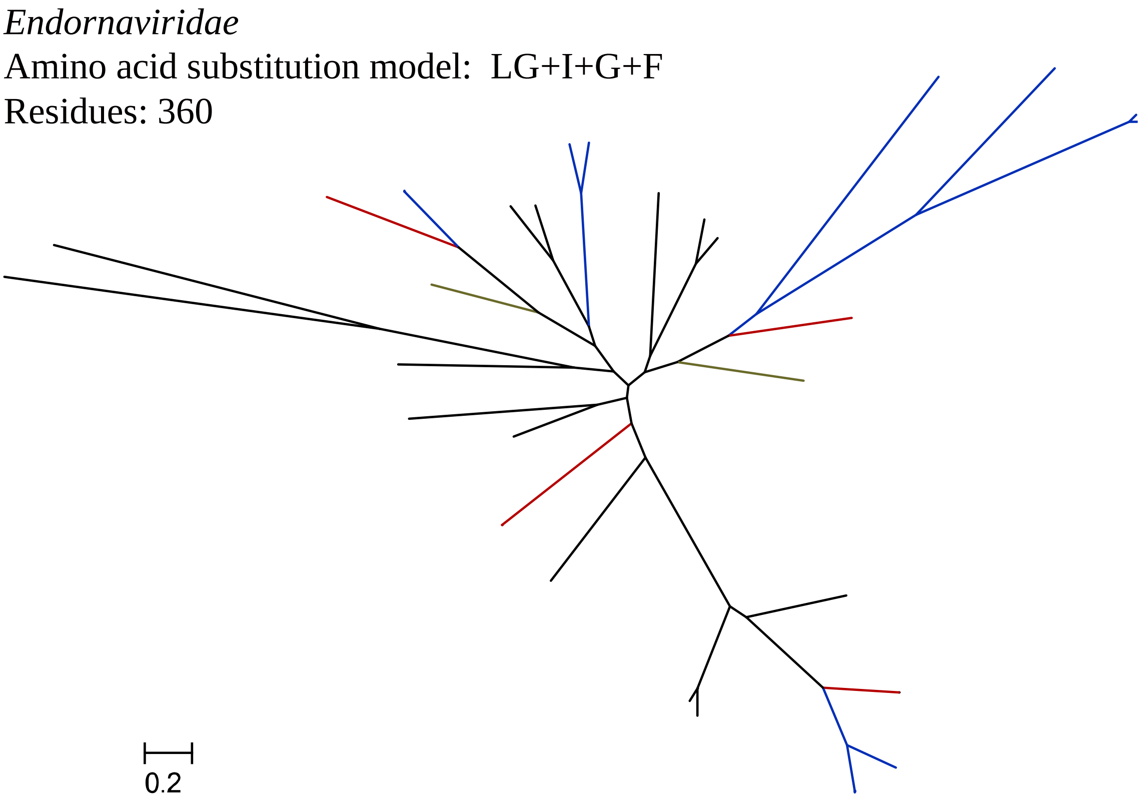
Endornaviridae
Amino acid substitution model: LG+I+G+F
Residues: 360
0.2

(F)

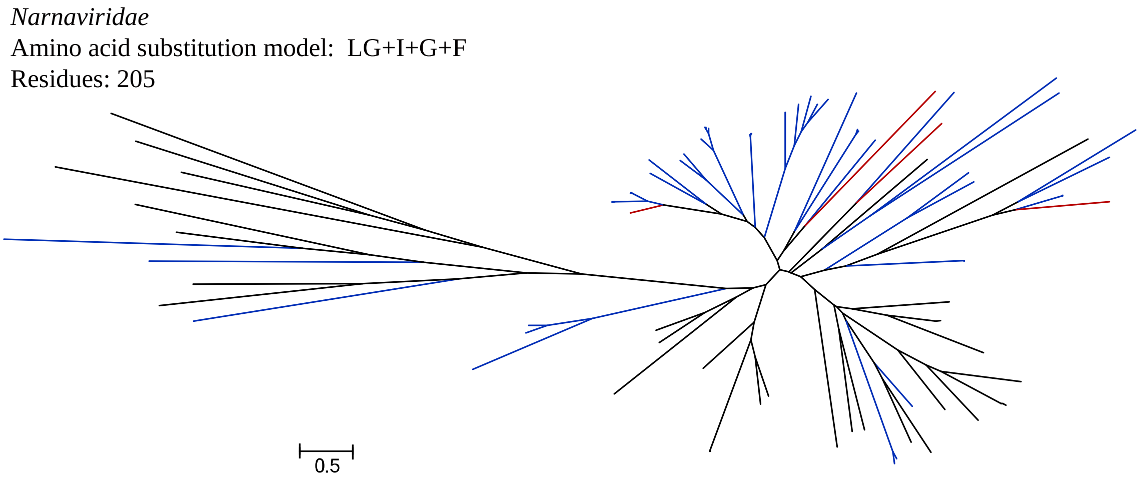
Narnaviridae
Amino acid substitution model: LG+I+G+F
Residues: 205
0.5

(G)

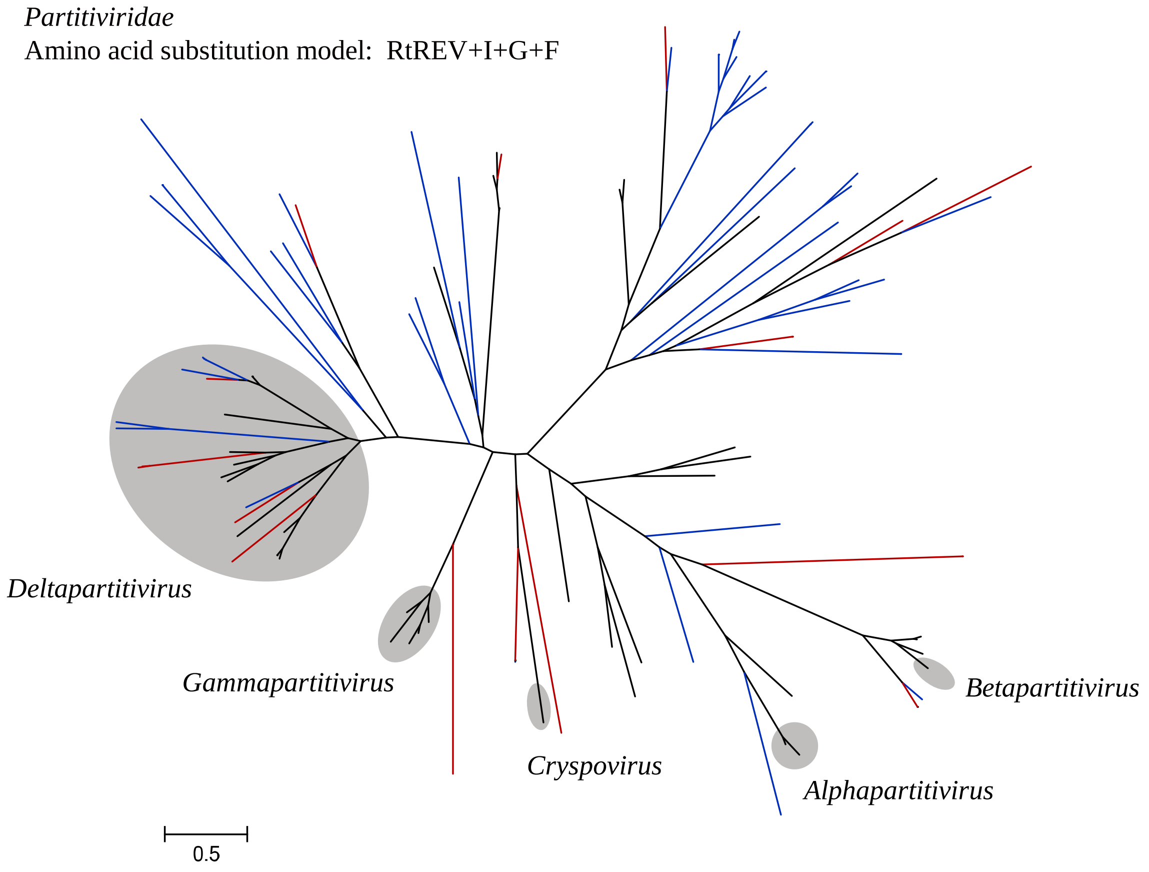
Partitiviridae
Amino acid substitution model: RtREV+I+G+F
Deltapartitivirus
Gammapartitivirus
Cryspovirus
Alphapartitivirus
Betapartitivirus
0.5

(H)

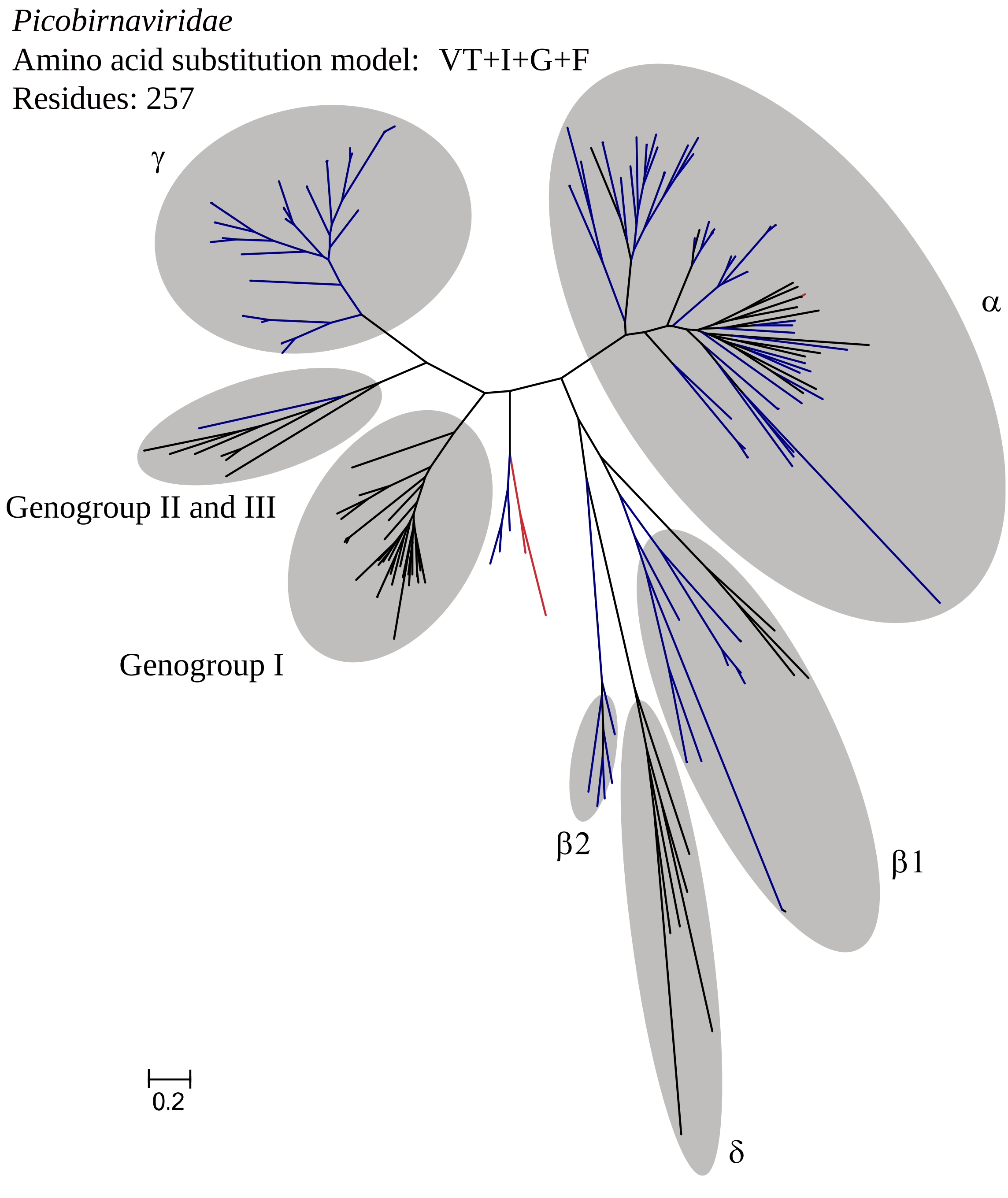
Picobirnaviridae
Amino acid substitution model: VT+I+G+F
Residues: 257
γ
α
Genogroup II and III
Genogroup I
β2
β1
δ
0.2

(I)

Reoviridae
Amino acid substitution model: VT+I+G+F
Residues: 236

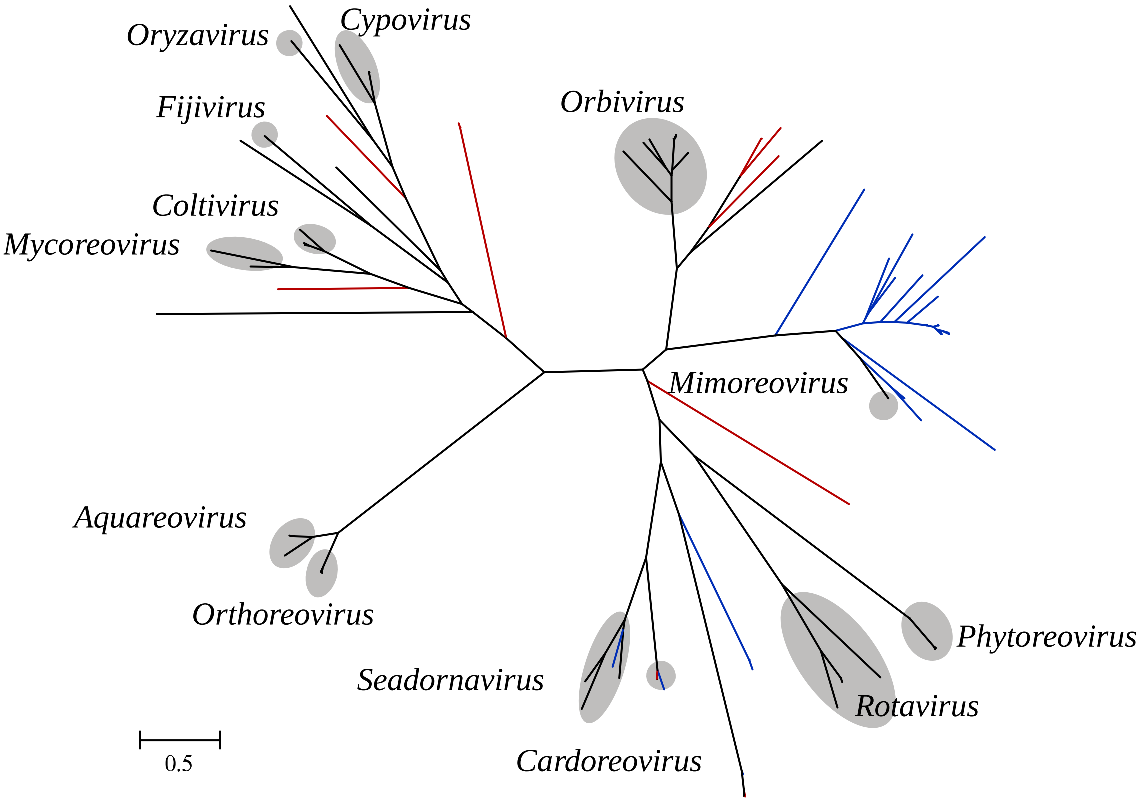

(J)

Totiviridae
Amino acid substitution model: LG+I+G+F
Residues: 227

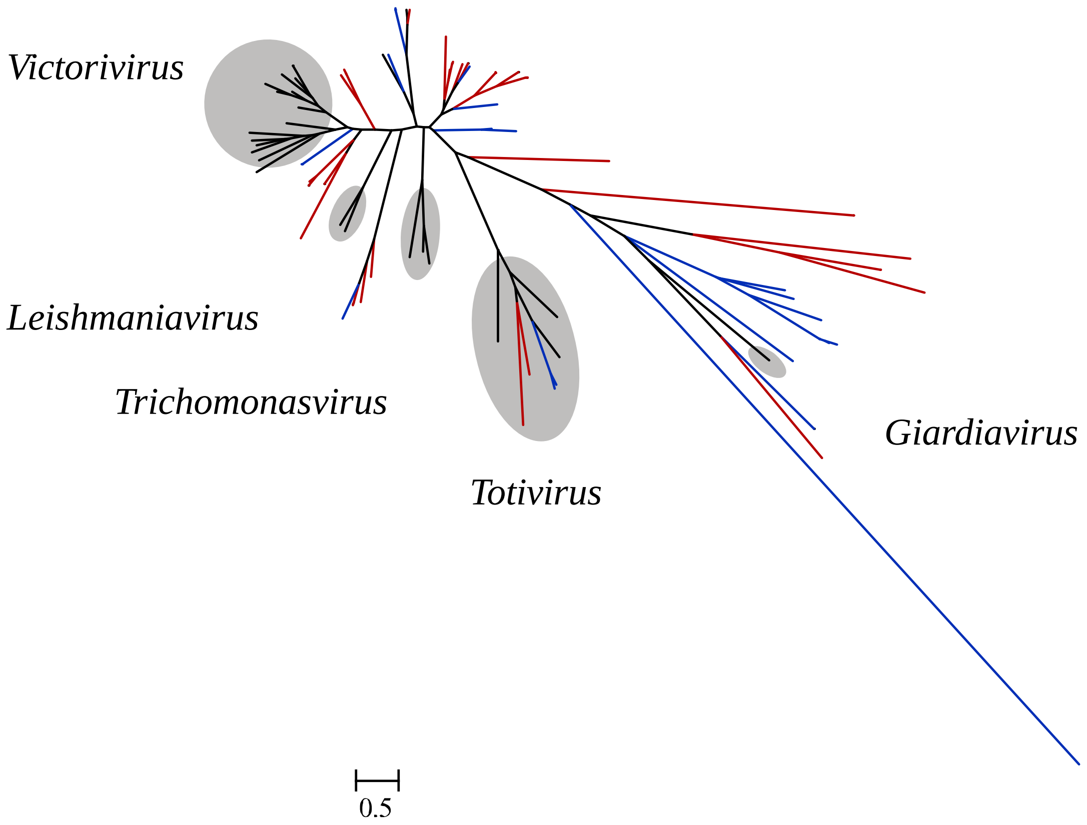

Fig. S6. Maximum likelihood trees of aligned RdRp amino acid sequences from minor or partial RNA virus contigs identified in this study (red lines) and representative members of previously reported RNA viruses in families (A) *Birnaviridae*, (B) *Chrysoviridae*, (C) *Cystoviridae*, (D) *Dicistroviridae*, (E) *Endornaviridae*, (F) *Narnaviridae*, (G) *Partitiviridae*, (H) *Picobirnaviridae*, (I) *Reoviridae* and (J) *Totiviridae*. Blue lines, brown

lines and green lines represent OTUs derived from Urayama et al. (2018b), related sequences identified from the Transcriptome Shotgun Assembly sequence database and our unpublished data, respectively.

Table S1. RNA viral contigs from the sponge.

Excel file

Table S2. BLASTP hit list of predicted ORFs.

Virus name	Segment or ORF	Top hit protein	Accession	e-value	Virus	Family
SdRV	ORF1	putative ORF1	ASM93984.1	0.0	Caledonia beadlet anemone dicistro-like virus 2	*Dicistroviridae*
	ORF2	putative ORF2	ASM93985.1	0.0		
SrRV	RNA1	RdRp	AVM87459.1	2e-89	Wenling scaldfish reovirus	*Reoviridae*
SpaRV	RNA1	RdRp	APG78297.1	2e-43	Hubei partiti-like virus 53	unclassified
SpiRV	RNA2	RdRp	APG78305.1	2e-106	Hubei picobirna-like virus 2	unclassified
